# Ubiquitous Non-Wearable Sensor for Human Sedentary Behavior Monitoring and Characterization

**DOI:** 10.3390/s26082468

**Published:** 2026-04-17

**Authors:** Anjia Ye, Ananda Maiti, Matthew Schmidt, Scott J. Pedersen

**Affiliations:** 1 School of Education, University of Tasmania, Launceston, TAS 7248, Australia; scott.pedersen@utas.edu.au; 2 School of IT, Deakin University, Waurn Ponds, VIC 3216, Australia; 3 School of Health Sciences, University of Tasmania, Sandy Bay, TAS 7005, Australia; matthew.schmidt@utas.edu.au

**Keywords:** sedentary behavior, edge computing, human activity recognition, workplace health, Internet of Things (IoT)

## Abstract

**Highlights:**

**What are the main findings?**

- The privacy-preserving sensor array accurately classified the number and the timeframe of sitting, standing, and away periods in a desk-based environment.
- Proposed architecture differentiates between sitting, standing at a desk and physical absence while achieving 100% sensitivity for desk-based exercise repetition tracking.

**What are the implications of the main findings?**

- A light-weight privacy-preserving sensor is capable of accurately detecting human behaviors and modifying movement prompt timing to reduce sedentary time and improve health.
- Real-time behavior recognition enables automated exercise logging, eliminating the human bias and burden inherent in self-reported compliance with exercise.
- The proposed sensor mechanism demonstrates the feasibility necessary to be integrated into a natural environment, as tested in an office.

**Abstract:**

Occupational sedentary behavior presents a public health risk, yet current interventions often rely on subjective self-reports or context-blind prompts. This study validates a privacy-preserving, edge-computing time-of-flight (ToF) sensor that detects postural states and quantifies therapeutic exercise gestures in real time. The dual-sensor architecture distinguishes between sitting, standing, and absence, while capturing rapid sit-to-stand repetitions suitable for active-break interventions. In this paper, a laboratory study (*N* = 7) evaluated the system against ground truth comprising activPAL3 accelerometry and video analysis. Across 378 postural events, the sensor achieved high temporal fidelity (mean absolute error < 1.6 s) and 100% sensitivity in counting exercise repetitions. The system differentiated workstation occupancy from physical absence. These findings demonstrate that ToF sensing matches the accuracy of video analysis without privacy concerns while offering the contextual awareness required for just-in-time, adaptive workplace interventions.

## 1. Introduction

Sedentary behavior, defined as any waking behavior characterized by an energy expenditure of less than 1.5 metabolic equivalents while in a sitting, reclining, or lying posture, has emerged as a critical global public health challenge. Systematic reviews consistently report that office workers spend the most time in sedentary behavior among occupational groups, often spending a disproportionate amount of their workday seated compared to other professions [1,2]. This accumulation of prolonged, unbroken sedentary time is strongly associated with an increased risk of all-cause mortality, cardiovascular disease, and type 2 diabetes [3]. Furthermore, occupational sedentariness negatively impacts mental well-being, contributing to heightened stress, fatigue, and an increased risk of depression [4,5]. Consequently, developing effective strategies to interrupt prolonged sitting at the workplace is key to reducing office-based sedentary behavior and mitigating the associated negative health outcomes.

Despite these documented risks, modifying behavior in office environments remains difficult. A significant limitation in current workplace health research is the heavy reliance on self-report tools, such as surveys and logs, to evaluate intervention efficacy [6,7]. While cost-effective, self-reported data are highly susceptible to recall bias and social desirability effects, often resulting in the overestimation of physical activity and the underestimation of sitting time [8,9]. This measurement inaccuracy leads to weak or inconsistent evidence regarding the true impact of health interventions. To robustly evaluate behavioral change and deliver timely prompts, interventions require objective data that can accurately distinguish sedentary from active states in real time.

However, integrating objective monitoring into workplace interventions presents significant logistical and ethical hurdles. While wearable sensors and optical cameras offer high precision, they face substantial barriers regarding user compliance, battery management, and privacy concerns in professional environments [10,11]. Without a non-intrusive method to objectively detect user presence and posture, digital interventions remain “context-blind,” frequently triggering prompts when users are already active or absent. To address this, this study presents a privacy-preserving, desk-based sensor architecture using ToF technology. By processing data locally, the system aims to facilitate responsive, context-aware health interventions that adapt to the user’s actual behavior.

In addition to modifying the user’s sedentary behavior, we aim to develop an exercise routine for the user and monitor their performance as they follow it. The primary motivation for a dedicated desktop-based sensor is to both passively monitor human users for sedentary behavior and actively monitor an activity the user has been asked to perform within a time limit. Both of these can be done with the same device, and this requires design flexibility. The first type of monitor is passive, slow, and largely varies with the nature of human users. The second type of monitoring is similar to gesture recognition in human–computer interaction (HCI), as it must occur quickly and in a specific manner.

This research work is based on a laboratory trial with the proposed sensor [12]. The use of such a sensor system within a workplace health intervention aims to modify the timing of movement prompts. Specifically, the system detects self-initiated ‘*stand*’ and ‘*away*’ events to automatically postpone scheduled movement and exercise prompts. This would create a more responsive and less intrusive user experience by recognizing natural movement breaks and prompting the user to move only after prolonged sitting.

While the sensor system has been technically proven to be efficient to set up and operate at a limited prototype level [13], a key remaining challenge is to demonstrate its ability to effectively monitor users of varying heights and movement characteristics. In this study, a semi-scripted movement schedule was used to provide the participants with an immersive experience, allowing them to behave naturally in front of the sensor and express their natural movement patterns. The script ensured that sufficient actions with varying delays were captured to assess the device’s sensitivity and error rates. The key contributions of this paper are as follows:

- A sensor system that uses differential distance data to detect and quantify specific desk-based presence and physical activities. It demonstrates the ability to count repetitions during vertical-displacement exercises, such as desk squats, transforming the workstation into an active platform for monitoring “just-in-time” exercise interventions.
- The proposed sensor’s validity against a gold-standard video ground truth. The validation evaluates performance across a range of temporal resolutions, confirming detection accuracy for both metabolically meaningful sedentary bouts (exceeding 60 s) and rapid postural transitions typical during repeated exercises (<3 s).
- An in-depth analysis of the sensor’s performance of both typical office behaviors and active gesture recognition monitoring during exercise. The sensor accuracy and classification of standard desk-based behaviors of sit, stand, and away were potentially challenged by the higher variability of the participants performing these commonplace behaviors. Conversely, classification accuracy was higher for participants performing repeated exercises, which were putatively less variable.

The remainder of the paper is organized as follows: Section 2 discusses previous work in sedentary behavior monitoring, the sensing technologies used, and the different paradigms of sedentary behavior monitoring and gesture recognition in the field of HCI. Section 3 presents the device architecture, and Section 4 discusses the laboratory trials process. The results and the corresponding discussion are presented in Section 5 and Section 6.

## 2. Related Works

### 2.1. Sedentary Behavior Problem: Exertime/Barrier

To address occupational sedentariness, various e-health interventions have been deployed. One example is Exertime™, a software-based intervention designed to interrupt prolonged sitting. Early evaluations demonstrated clinical efficacy; pilot studies indicated that passive computer prompts were effective in promoting adherence to movement breaks [14], while randomized controlled trials showed reductions in mean arterial pressure and job-related stress among employees utilizing the software [15,16].

Despite these physiological benefits, recent qualitative evaluations have identified critical barriers to long-term adoption. Carter et al. [17] reported that users frequently perceived the software’s prompts as “aggressive” and “intrusive.” A primary source of friction was the “lock-out” feature, which forcibly took over the screen regardless of the user’s workflow. Similarly, Oliver et al. [18] found that such forced interruptions were operationally unsuitable for high-risk environments, such as police control rooms. A recurring theme in these critiques is the software’s lack of autonomy and context awareness; users reported frustration when prompts triggered while they were already standing, teaching, or away from their desks [18]. This “context-blindness” forces users to engage with the software unnecessarily, degrading trust and adherence.

### 2.2. Detecting Sedentary Behavior

To mitigate the intrusiveness of these software interventions, a passive, automated interaction method is required. This necessity drives the research towards directly sensing movement. Previous studies have attempted to use wearable sensors, such as the activPAL, to validate Exertime’s built-in exercise logs, revealing discrepancies between self-reported compliance and actual activity [8]. However, translating wearables into permanent workplace interventions is challenged by the burden of adherence, including device charging, skin irritation, and the logistics of deploying devices to large workforces [11]. Furthermore, wearables typically lack spatial context; they cannot easily distinguish between a user standing at their desk and one who has walked to a different location [19].

Ambient sensors offer a non-wearable alternative. Technologies such as pressure mats, instrumented chairs, and ultrasonic sensors have been utilized to detect user presence [20,21]. However, while devices like pressure mats accurately detect sitting time, they typically fail to differentiate between a user standing at the workstation and one who has vacated the area [22]. Additionally, maintaining the battery can be challenging during long-term deployments. While optical cameras and computer vision provide high-fidelity posture analysis [10], they are widely rejected in office settings due to surveillance risks [23,24]. This concern is corroborated by our preliminary surveys and interviews with Exertime users, which indicated a high level of privacy awareness and a strong preference for non-identifiable monitoring solutions over cameras. Consequently, there is a gap for a privacy-preserving, non-wearable sensor that can provide granular, real-time data to drive intelligent workplace health interventions.

### 2.3. Human–Computer Interface—Detecting User Behavior

In HCI, passive sedentary behavior monitoring and gesture recognition represent distinct interaction paradigms that differ in user intent, temporal scale, and system responsiveness.

Passive monitoring involves continuously observing a user’s physical states or behaviors without requiring explicit interaction, often leveraging wearable or environmental sensors to infer long-term physical activity patterns such as sitting or inactivity for health or context-aware applications (e.g., objective sedentary behavior measurement using wearables) [25]. In contrast, gesture recognition is an active, short-duration interaction in which users deliberately perform specific movements (e.g., hand or body gestures) that the system interprets in real time to control digital interfaces or trigger commands. Research demonstrates gesture recognition for HCI using visual, inertial, or multimodal sensing modalities [26].

#### 2.3.1. Passive Sedentary Behavior vs. Gesture Recognition

From an HCI design perspective, passive sedentary behavior monitoring prioritizes low user burden, unobtrusiveness, and the delivery of contextual insights over prolonged periods. The system operates largely in the background, interpreting sensor data (e.g., from wearables or smartphones) to model users’ activity patterns, often for health feedback or for adaptive systems that support well-being without requiring explicit user actions. Here, interaction is indirect and analytic, with evaluation metrics focusing on the reliability of long-term inference, ecological validity, and user comfort. *Ethical* and *privacy* considerations also play a central role, given the continuous nature of data collection and the potential sensitivity of inferred behavioral states.

By contrast, gesture recognition in HCI is oriented toward intentional, real-time control, in which users’ dynamic or static movements serve as direct input signals. Research in gesture recognition for HCI explores both vision-based systems and wearable-sensor approaches that enable systems to detect, classify, and respond to gestures with low latency, enabling natural, contactless interaction paradigms in applications ranging from sign language interpretation to immersive environments and IoT control. In this context, system responsiveness, accuracy, and user feedback are critical to usability; errors are immediately apparent and can undermine the interaction experience if the system misinterprets user intent. Thus, gesture recognition demands fine-grained sensing and robust pattern recognition models to support seamless user control.

#### 2.3.2. Differences in Monitoring Techniques

Researchers have employed a range of sensing and computational approaches to detect and quantify sedentary behavior primarily for health and activity assessment. Common sensor modalities include wearable inertial sensors (accelerometers, gyroscopes, magnetometers) placed on the wrist, trunk, or thigh to capture posture and movement patterns [25,27,28]. These raw signals are then processed to extract features (e.g., counts, orientation, energy measures) and are fed into machine learning classifiers such as support vector machines (SVM), k-nearest neighbors (KNNs), Naive Bayes, or neural networks to distinguish sedentary from active states with high accuracy (~90%) in studies using smart garments or smartphone inertial measurement units (IMUs) [28].

For example, Sinha et al. used correlation-based feature selection and particle swarm optimization to select informative features from accelerometer and gyroscope data before SVM classification to recognize sitting behaviors (accuracy of ~99.9%) [29]. In addition to discriminative models, traditional cut-point algorithms based on thresholds of acceleration magnitude (e.g., mean amplitude deviation) are often applied to estimate sedentary time from continuous motion streams, particularly in epidemiological studies [30]. Other approaches include posture sensors (e.g., goniometers, pressure sensors) and vision-based depth data (e.g., Kinect-derived sit-to-stand detection) that infer sedentary behavior by analyzing body orientation and transitions over long periods [31].

Gesture recognition for interactive control draws on a rich set of sensing technologies and computational frameworks to identify intentional movements in real time. Traditional vision-based methods use cameras and computer vision algorithms (e.g., background subtraction, Otsu thresholding, contour tracking, articulation/pattern templates) to segment and track hands and bodies, then classify gestures via feature extraction and pattern matching [32].

Wearable and device-free modalities include IMUs on wrists or gloves, where time-series patterns of acceleration and rotation are processed via machine learning (e.g., SVM, random forests) or time-series analysis techniques like recurrence quantification analysis (RQA) to detect gesture onset and class [33]. Deep learning approaches (e.g., convolutional neural networks or recurrent architectures) are increasingly used to directly learn spatio-temporal features from raw data (e.g., radar or RGB/optical inputs) for complex gesture sets, enabling greater robustness and generalization [34]. Non-vision sensing alternatives, such as acoustic (MFCC/feature extraction) classifiers and infrared/reflected light wave signals, have also been demonstrated to recognize hand gestures by extracting discriminative signal features and classifying them with SVM or similar models [35].

The key differences in monitoring techniques for active (gesture recognition) and passive (sedentary behavior) can be summarized as follows:

◦ *Temporal scale and latency*: Sedentary behavior methods emphasize longer windows, summarizing motion or posture over minutes to hours, while gesture recognition focuses on short, real-time windows for immediate interaction.
◦ *Feature patterns*: Sedentary detection often relies on statistical and threshold-based movement summaries and relatively simple classifiers, whereas gesture recognition frequently uses rich spatio-temporal features and advanced models (e.g., deep networks) to capture fine movement dynamics.
◦ *Sensor choice*: Wearables (IMUs, heart rate, pressure) and depth/smartphone sensors dominate sedentary sensing; gesture recognition spans vision, inertial, acoustic, radar, and light-based sensors, often fused for robustness [36].

From a computational and HCI standpoint, *gesture recognition* is generally the more technically challenging problem than sedentary behavior monitoring [26,37,38]. This is due to gesture recognition operating under strict real-time constraints that require accurate segmentation, classification, and feedback within milliseconds; even small errors are immediately visible to users and can severely degrade usability. The variability of gestures across users, contexts, viewpoints, and execution styles further complicates modeling.

However, the primary aim of the sensors in this work is health and exercise, and any *gestures* are intended to be integrated into the user’s schedule. This means the start of the activity can be externally timed, thereby improving temporal awareness when classifying. So, we include active monitoring of an exercise to establish the impact of human intent and focus on the accuracy of state or event detection, if any. Hence, we chose an exercise that is relatively simple and involves similar steps to those in sedentary behavior monitoring. This reduces the traditional risks associated with obtaining gesture recognition and, for this paper, avoids the need for additional analysis to demonstrate the device’s accuracy.

In contrast, passive sedentary behavior monitoring benefits from temporal averaging and tolerance to short-term errors: misclassification durations on the order of seconds will cancel out over the long sensing duration, which is usually hours. However, it is still desirable to capture the new state or event as soon as possible. Also, human behavior is variable and may be more so in a natural desk-based environment than in repeated performance of the same task. This variability could directly result in failure to detect events, as well as potential system misconfigurations and continuous external noise. However, sedentary monitoring introduces methodological challenges, including privacy concerns, sensor drift, long-term robustness, and ambiguity between similar non-interaction-critical postures.

Current monitoring technologies do not meet the operational requirements for continuous workplace health interventions. Wearable devices require daily maintenance and lack spatial context; they cannot distinguish between a user standing at a workstation and one standing elsewhere. Optical cameras capture spatial context but introduce privacy risks, preventing their deployment in office environments. Alternative environmental sensors, such as pressure mats, detect seated postures but cannot quantify upright activity or dynamic exercise.

A research gap therefore persists: no available system provides context-aware, non-wearable, and privacy-preserving measurement for monitoring long-term sedentary behavior. To address this gap, this study develops and validates a dedicated sensor architecture. The proposed system operates continuously without requiring user adherence, passively monitors spatial occupancy, and actively quantifies prescribed movements. Furthermore, objective data generated by human activity recognition can replace traditional self-reporting used in workplace health interventions. By automatically logging completed exercises and postponing scheduled prompts based on recognized behavior, this architecture supplies the real-time data required to drive adaptive, just-in-time workplace health interventions.

## 3. Device Architecture and System Design

### 3.1. Privacy-Preserving Edge Architecture

The development of the sensor system was governed by the comprehensive integration of privacy preservation, ease of use, and cost-effectiveness. A top priority was protecting user data in the workplace. Unlike previous proof-of-concept designs that relied on a cloud-centric architecture where real-time distance data were streamed to a central server, the current system was re-engineered as an edge computing device [12]. This shift was driven by user surveys and interviews indicating significant apprehension regarding the transmission and storage of raw behavioral data on external servers [22]. To address this, a custom web browser extension was developed to perform all data processing locally on the user’s end. When a valid movement break is detected, the extension communicates only an instruction to the server, effectively logging the activity and/or resetting the prompt timer without compromising data privacy.

To ensure long-term adherence, the system was further designed to impose a negligible burden on the user. While wearable devices often suffer from attrition due to the requirement for active user participation, such as daily charging and physical attachment, this desk-based solution is powered directly by the workstation via USB and operates autonomously. This passive data-collection model ensures seamless monitoring without disrupting the user’s workflow or requiring behavioral changes.

**Figure 1:**
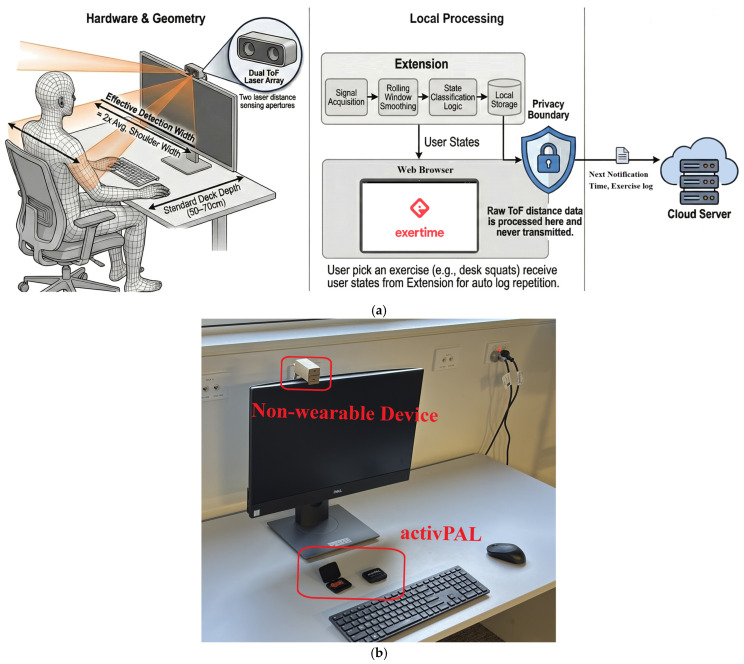
(**a**) System architecture and privacy-preserving data flow; (**b**) actual sensor mounted on screen and AP3.

Finally, to facilitate scalability, the hardware architecture was streamlined to utilize a single ToF array comprising two sensors [22,24]. The housing device was also designed using sustainable, recyclable cardboard, distributed as a flat-pack kit, thereby reducing manufacturing and shipping costs and potentially increasing user engagement during assembly (Figure 1). The angle between the sensors is fixed during installation and adjusted to the user’s height and workstation conditions. It is configured such that one sensor points towards the user’s torso while seated, and the other goes just over their head to infinity (or a very large distance). When the user is standing, both sensors would return finite values.

### 3.2. Differential Distance Classifier 

**Figure 2:**
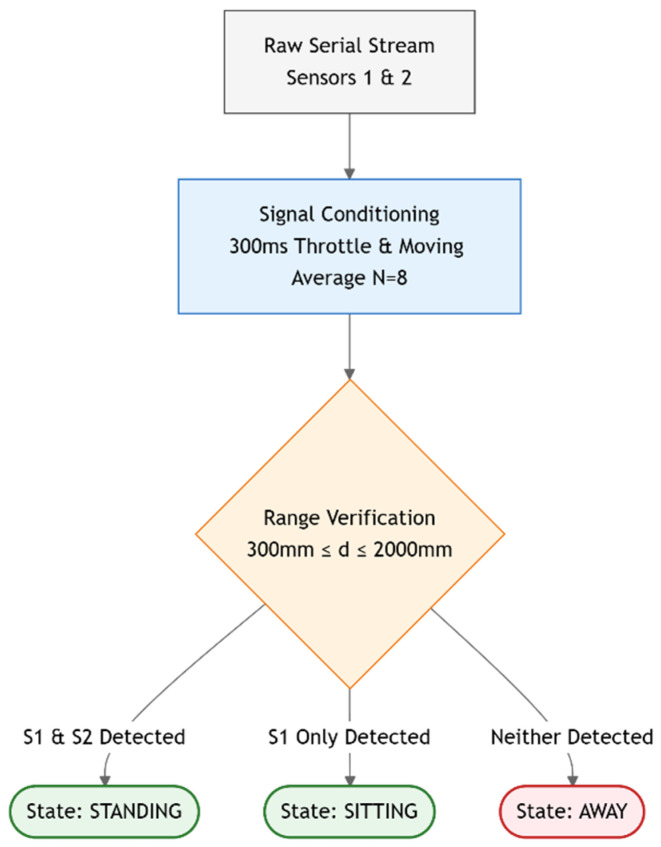
Flowchart of the Differential Distance Classifier algorithm.

Raw distance inputs from sensors aimed at the seated torso and just above the seated head are first processed with a moving-average filter to smooth signal spikes caused by transient sensor noise. Subsequently, a decision logic gate determines the candidate state based on valid thresholds within the range of 300 mm to 2000 mm. Using a decision tree structure, the system classifies the user’s state as ‘sitting’ if only the torso is detected within range, ‘standing’ if both the torso and head are detected, or ‘away’ if neither sensor detects a presence. Finally, to align with clinical guidelines for healthy nudges, a minimum duration threshold of 60 s was applied to ‘stand’ and ‘away’ events. Recent evidence indicates that activity bouts require a minimum duration of one minute to yield positive cardiometabolic outcomes; consequently, transitions shorter than this window were excluded to ensure that the system logs only the behavior relevant to effective interventions (see Figure 2). The 60-s parameter serves solely as a trigger for the intervention and does not affect the Differential Distance Classifier’s functionality.

The operational parameters for this heuristic baseline were empirically selected to balance signal stability with physiological relevance. For signal conditioning, a window size (ω = 8) stabilized the “no detection” state (sensor maximum: 8191 mm). Preliminary testing indicated that a smaller window (ω = 4) caused excessive variance, dropping the idle signal to approximately 6154 mm; increasing the window to ω = 8 maintained a stable baseline above 7172 mm. Although this introduces approximately 2 s of processing latency, this delay is acceptable for monitoring static postural states.

For state classification, the range was defined as 300–2000 mm. The 300 mm lower bound accommodates users leaning forward, preventing data clipping at distances closer than the standard 60–80 cm viewing distance, while the 2000 mm upper bound captures backward leans (typically up to 1800 mm) and rejects background movement (e.g., co-workers passing by).

## 4. Methods

A laboratory-based validation study was conducted to determine the criterion validity of the novel sensor’s ability to detect and measure desk-based postural events. A purposive sample of healthy office workers (*N* = 7) with heights ranging from 160–190 cm (mean 176 cm) was recruited to reflect a variety of body heights and sitting positions. The study received approval (H0028408) from the University of Tasmania Human Research Ethics Committee. All participants were provided with a detailed information sheet and gave written informed consent prior to data collection. Participants were informed of their right to withdraw at any time and could request that their data be deleted up to two weeks after collection.

### 4.1. Instrumentation and Data Acquisition

Data acquisition involved three simultaneous streams to capture participant posture and movement. The test measure consisted of a device comprising two adjustable ToF sensor arrays housed within a single unit. One sensor was oriented downward toward the participant’s chest, while another was oriented upward above seated head height; postural changes were identified by significant shifts in distance measurements from one or both sensors. An onboard Arduino Nano microcontroller timestamped and recorded all sensor readings to a host workstation equipped with an Intel^®^ Core™ i7-8700 CPU @ 3.20 GHz and 16.0 GB of RAM. The distance was recorded at 3.3 Hz.

To establish criterion validity, two reference measures were employed concurrently. First, participants wore an AP3 micro (PAL Technologies, Glasgow, UK) on the anterior midline of the right thigh. Widely recognized as a criterion measure for assessing lab- and free-living sedentary behavior (e.g., [11,22,39]), the device sampled raw acceleration data at 20 Hz. For analysis, data were processed with a minimum sitting or upright period of 1 s in PAL analysis (v9.1.0.102), enabling the detection of high-resolution postural shifts. Second, a web camera positioned 5 m from the workstation captured the participant’s full body and the immediate desk environment. The camera was programmed to capture timestamped still images at 2 frames per second (fps). This sampling rate provided double the temporal resolution of the AP3’s 1-s event epoch, ensuring that rapid transitions could be visually verified with sub-second precision.

### 4.2. Experimental Protocol

Following instrumentation, participants performed three scripted protocols (see Appendix A) at a workstation setup in the laboratory. Instructions were delivered via on-screen prompts with audio guidance programmed by the researcher.

- *Protocol 1* measured static postural duration (sitting and standing) using bouts ranging from 60 to 150 s.
- *Protocol 2* evaluated user presence (sitting and standing) versus absence (away) by incorporating desk-departure periods of 60 to 150 s.

In both protocols 1 and 2, we let the user relax and perform regular activities without focusing on the sensor. The user immerses themselves in typical desk-based tasks. We created a movement script to ensure we collect enough data. Users are prompted for the next action at specific intervals, e.g., to sit, stand, or move away. While users are aware of the activity in the lab trial, they do not focus on the sensor and can perform the movement in a relaxed manner to imitate a real office desk. We compare the ground truth of actual movements with sensor data to account for any user deviation from the script.

- *Protocol 3* validated the logging accuracy of a gamified health intervention; participants performed 10 desk-based squat cycles (20 discrete sitting-to-standing events) at a cadence of 6 s per cycle to test sensor performance during rapid movement.

The desk squat is a *gesture* of rapid succession of sit-to-stand transitions within a short, fixed period. This is a time-limited activity in which the user is asked to perform the exercise with the intention of doing so and is conscious and focused on the sensor during the act.

### 4.3. Data Analysis 

#### 4.3.1. The Ground Truth

Immediately prior to data collection, the clocks on the two workstations with the webcam and ToF sensor were synchronized. The system embedded these timestamps as file names for the captured video frames. The AP3 used the same time set by the PAL connect (v9.1.5.179) app on the same workstation, just before commencing the trials.

A pilot data-collection and analysis session demonstrated that the AP3 overlooked brief standing or stepping events and failed to record absolute location. As a result, it failed to distinguish between away and stand events and could not be used as the gold standard by itself. To address these inherent limitations and increase analytical efficiency, the study adopted a hybrid ground-truth approach: For protocol 1, AP3 data were cross-referenced against script timestamps, and only instances with discrepancies exceeding 5 s were manually reviewed to generate the definitive ground truth. The ground truth for Protocol 2 relied exclusively on camera data as the reference standard because AP3 misclassified ‘away’ events as upright time and Protocol 3 events as stepping time. Protocol 3 was validated against the exercise log using the scripted protocol.

#### 4.3.2. The Distance Time Series

The sensor recorded distance data at 3.3 Hz (300 ms per interval). The system applied the Differential Distance Classifier immediately after recording a data point, resulting in a sequence of states $A=\left\{sit,\;stand,\;away\right\}$. When the user moves, the state changes and is retained in subsequent time stamps until the next change. This is used to create another dataset that records only the timestamp and the new state whenever it changes. Similarly, we can obtain a timestamp corresponding to an event (i.e., the change in state) in the ground truth.

From this, for a user *u*, we obtain a set of sensor-detected timestamps *t_i_*, denoted *S* = {*e_1_*, *e_2_*, …}, and a corresponding set of ground-truth timestamps *t_i’_* representing the actual physical actions, denoted *G* = {*e_1_’*, *e_2_’*, …}. To evaluate performance, we align these datasets by pairing each sensor timestamp in S_u_ with its nearest-neighbor timestamp counterpart in *G_u_.* This is done by aligning the *t_i_’* with *t_i_* where *t_i_*-*t_i_’* is lowest. This may leave a gap in the sensors’ timeline if an event was completely missed by the sensor. Eventually, *t*his means that if an *event* is missed, the preceding *event* is recorded as longer than it actually was, i.e., including the duration of the missed *state* or *event*.

To evaluate the sensor’s utility for occupational health interventions, the statistical analysis adopts the validation methods established by Ryde et al. [22] for objective measures of occupational sitting. While system latency is an inherent hardware characteristic, the primary criterion for clinical validity is the accuracy of the recorded event duration and the correct quantification of accumulated actions. Therefore, rather than characterizing raw latency, we assessed the agreement between the sensor and ground truth regarding the temporal magnitude of actions.

Also, the duration of each action can be derived from the interval between consecutive transition timestamps. For a specific event i, the duration $D_{i}$ is calculated as the difference between the timestamp of the current event ($t_{i}$) and the timestamp of the subsequent event $\left(t_{i+1}\right):$

(1) $$D_{i}=t_{i+1}-t_{i}$$

This calculation is performed independently for both the sensor-detected sequence (*S_u_*) and the ground-truth sequence (*G_u_*), enabling a direct comparison of the measured durations for each discrete postural bout.

#### 4.3.3. Minimum Acceptable Transition Detection Thresholds

The fundamental parameter for evaluation is the delay threshold, δ, defined as the absolute temporal difference between the ground truth timestamp for $\left(e_{i}^{\prime }\right)$ and the sensor timestamp for $\left(e_{i}\right)$ for a specific event transition:

(2) $$\left|t_{i}^{\prime }-t_{i}\right|\leq \delta$$

For applications involving real-time feedback, δ cannot be infinite. It must remain below the maximum tolerance ($e.g.,\;\delta_{T}=5\;s$), a limit determined by hardware hysteresis and typical human reaction speeds.

For Protocols 1 and 2, which assess the detection of sustained postures (sit, stand, away), sensitivity ($\phi_{\delta }$) is defined as the system’s ability to correctly classify a state transition within the allowable delay:

(3) $$\phi_{\delta }=\;\left(\frac{\text{Number of correctly detected events within}\;\delta }{\text{Total number of ground truth events}}\right)\times 100$$

Post-analysis identified the optimal operational parameter by incrementing δ from 1 s to 10 s with the raw dataset. This process determined the *minimum acceptable delay threshold* ($\delta_{min}$) defined as the value at which detection accuracy plateaus while satisfying the constraint $\delta \leq \delta_{T}$. The *δ_min_* value indicates the minimum time required for a majority of random passive events to be detected by this sensor.

As Protocol 3 is a different type of activity, i.e., a gesture, there may be additional measures to ensure correctness. It involves a gesture composed of rapid, cyclic transitions (desk-based squats). Consequently, we can shift focus from temporal alignment to repetition counting and evaluate the capacity to capture high-frequency alternating cycles. The measure of correctness is therefore

(4) $$\psi =\left(\frac{\text{Number of detected repetition cycles}}{\text{Total number of scripted cycles}}\right)\times 100$$

Apart from the device’s sensitivity, other measures include the timeliness of detection and comprehensive validation, which assesses the quality and consistency of the detected data.

#### 4.3.4. Detection Delay Error Metrics

We calculated error metrics exclusively for correctly identified events (True Positives aligned within the δ tolerance). We expanded upon Ryde et al.’s use of mean differences by calculating the mean absolute error (MAE). This metric captures the average magnitude of timing error per event and is applied to detected events where $|S-G|\leq \delta_{min}$. *n* denotes the total number of detected events across all users.

MAE with $\delta_{min}$ is

(5) $$\mathrm{MAE}=\frac{1}{n}\sum_{i=1}^{n}|S_{i}-G_{i}|$$

with 95% confidence interval (for mean difference):

(6) $$CI_{95\%}=\overset{ˉ}{d}\pm 1.96\cdot \frac{\sigma_{d}}{\sqrt{n}}$$

We also calculated the count bias, which indicates the total number of events that were missing. This ultimately would indicate whether the system over- or underestimates the frequency of prolonged sitting:

(7) $$Bias_{count}=N_{i}-N_{ground_{truth}}$$

#### 4.3.5. Classification Metrics

To evaluate the sensor’s ability to distinguish between sitting, standing, and physical absence. Performance was assessed using a one-vs-rest confusion matrix approach [40]. In this framework, a True Positive (TP) represents the correct identification of the target state, whereas a False Positive (FP) indicates the incorrect attribution of the target label to a different posture. A False Negative (FN) reflects the failure to detect an occurring state, and a True Negative (TN) signifies the correct rejection of non-target activities.

(8) $$Precision=\frac{TP}{TP+FP}$$

Second, recall (equivalent to sensitivity) quantifies the proportion of actual events successfully detected. High recall shows low risk in the underestimation of sedentary bouts (FN), thereby preserving the integrity of longitudinal health data.

(9) $$\mathrm{Recall}=\frac{TP}{TP+FN}$$

Third, specificity evaluates the capacity to exclude non-target states. This metric is critical for the ‘away’ class, ensuring the system does not erroneously log workstation occupancy during physical absence.

(10) $$\mathrm{Specificity}=\frac{TN}{TN+FP}$$

Finally, the *F*_1_-score calculates the harmonic mean of precision and recall. This composite metric provides a single performance indicator that balances the trade-off between missed detections and false artifacts.

(11) $$F_{1}=2\cdot \frac{\mathrm{Precision}\cdot \mathrm{Recall}}{\mathrm{Precision}+\mathrm{Recall}}$$

#### 4.3.6. Individual User Variance

To characterize the influence of human characteristics on sensor fidelity, error metrics were stratified by participants. While the standard MAE provides an overall performance analysis for the device, the MAE calculated for an individual participant can indicate how that user may be characterized. We define this as MAE(u) for a user *u*.

(12) $$MAE\left(u\right)=\frac{1}{n_{u}}\sum_{j=1}^{n_{u}}|S_{u,j}-G_{u,j}|$$

where $n_{u}$ represents the total number of detected events for the participant u, $S_{u,j}$ denotes the sensor duration for the $j^{th}$ event, and $G_{u,j}$ corresponds to the ground truth duration for that same event.

## 5. Results

We analyzed 378 distinct postural events across the seven participants. Protocol 1 yielded 119 events (56 sit, 63 stand), protocol 2 contributed 119 events (14 sit, 42 stand, 63 away), and protocol 3 added 140 rapid transitions (70 sit, 70 stand). This design ensured sufficient data density to assess sensor validity across varying durations and contexts.

### 5.1. Duration and Event Detection

Table 1 presents the performance metrics for event detection and duration measurement.

#### 5.1.1. Protocol 1 (Sit-Stand)

Protocol 1 (P1) was designed to simulate a passive, sedentary monitoring scenario in which users gradually lose focus on the sensor until a script explicitly prompts them to change posture. In this context, the sensor demonstrated high reliability in quantifying overall sitting and standing times. As shown in Table 1, the device detected the exact number of postural shifts (count bias = 0). However, strict timestamp matching yielded sensitivities of 78.6% (sit) and 81.0% (stand) at minimum delay thresholds (*δ_min_*) of 3 and 4 s, respectively. This indicates that while the system successfully captured all events, minor synchronization lags caused some transitions to fall just outside the strictest temporal tolerance windows. Despite this slight latency, the Mean Absolute Error (MAE) for matched events remained exceptionally low at approximately 1.5 s.

Protocol 1 (P1) is designed as a passive, sedentary monitoring mechanism, in which users are expected to forget about the sensor monitoring over time until they are manually prompted or reminded of their next movement according to the script. While this is not truly passive, it is more passive than protocol 3.

#### 5.1.2. Protocol 2 (Sit-Stand-Away)

Like P1, Protocol 2 (P2) served as a passive monitoring mechanism but introduced greater spatial complexity. Unlike wearable devices (e.g., AP3), which typically aggregate standing and leaving the desk into a single ‘upright’ category, the ToF sensor successfully distinguished between standing at the workstation and moving away from it. While negative count biases (−1 to −3) indicated a minor under-detection of total event frequencies, the correctly detected events demonstrated excellent sub-second temporal precision for non-seated states. The MAE for ‘stand’ (0.13 s) and ‘away’ (0.67 s) was notably lower than observed in P1. This improvement suggests that the user’s distinct physical absence provides a clearer signal change than the vertical shift between sitting and standing. To achieve optimal sensitivity, varying delay thresholds were required: a *δ_min_* of 2 s yielded a 90.5% sensitivity for standing, while sitting and away states required a 4-s *δ_min_* to achieve sensitivities of 57.1% and 83.9%, respectively.

Like P1, Protocol 2 (P2) is also designed as a passive, sedentary monitoring mechanism.

#### 5.1.3. Protocol 3 (Exercise)

Protocol 3 (P3) shifted from passive monitoring to an active gesture-recognition protocol, in which users maintained focus on the sensor during high-frequency interventions. The sensor functioned flawlessly in this active state. With 100% sensitivity and a zero-count bias, the device successfully logged every rapid sit-to-stand transition within a *δ_m_*_in_ of just 2 s. Unlike the static protocols, duration metrics (Mean Difference and MAE) were not computed for these events; the transient, cyclic nature of the exercise gestures precluded establishing the steady-state conditions required for duration analysis. Instead, these findings confirm the hardware’s robust ability to track active break interventions and repetition compliance, independent of the stability constraints imposed by static posture monitoring.

Protocol 3 (P3) is designed as an active gesture recognition protocol, in which users are expected to maintain focus on sensor monitoring.

Apart from sensitivity, the correctness of the desk-squat was also accurate at 100%, with all participants recorded as completing the 10 transitions from sit to stand and vice versa.

### 5.2. Classification Performance

**Figure 3:**
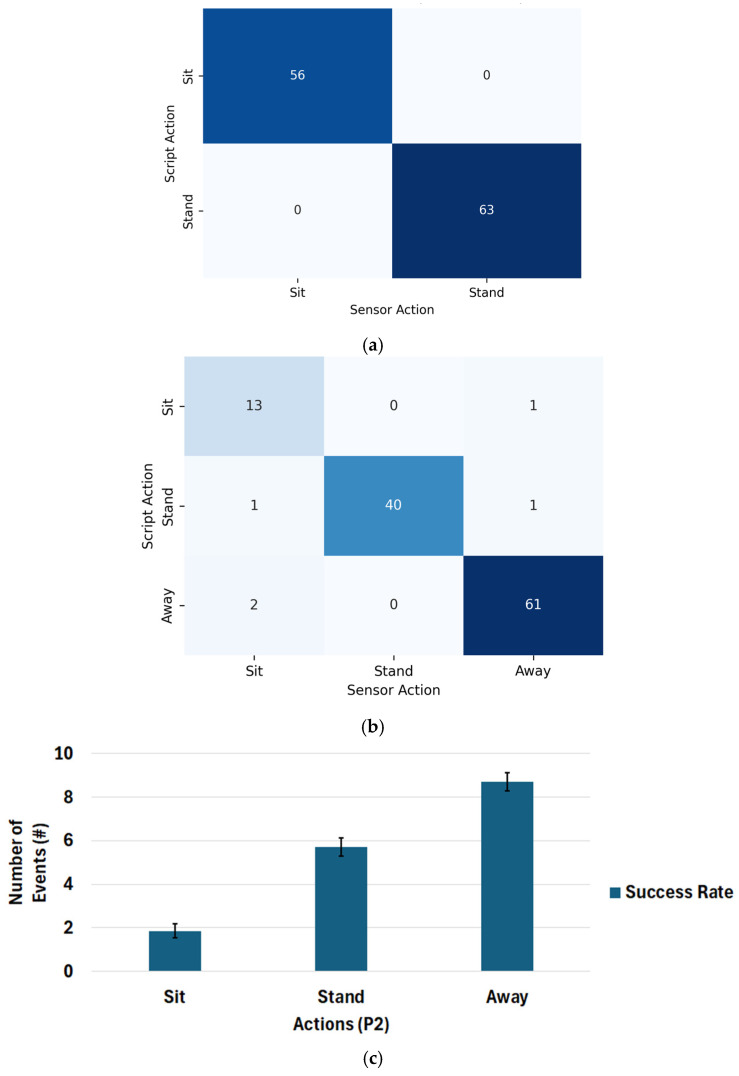
Confusion matrix for (**a**) protocol 1, sit, and stand; (**b**) protocol 2, sit, and stand away; (**c**) success rate in protocol 1 showing average detection rate for the participating users and 95% confidence.

Table 2 details the system’s classification performance. Figure 3 presents the confusion matrices detailing the absolute event counts for Protocol 1 and Protocol 2.

In protocol 1, the system achieved 100% across all metrics, detecting all 56 sit and 63 stand events. All 56 true scripted sit instances are correctly predicted as sit (no misclassification into stand). All 63 true stand instances are correctly predicted as stand (no misclassification into sit). The off-diagonal elements are zero, indicating no false positives or false negatives for either class.

The accuracy of the sit class in P2 was slightly lower than in P1, with 81.25% precision and 86.67% *F*_1_-score. As illustrated in Figure 3b, this drop occurred because the system missed one of the 14 actual sit events by misclassifying it as away, and generated false positives by incorrectly labeling one stand and two away events as sit. The stand class in P2 demonstrated 100% precision with a lower recall of 95.24% because two out of 42 events were missed (one misclassified as sit, one as away). Finally, the away state was correctly identified in 60 of 62 events (96.83% *F*_1_-score) with only two instances misclassified as sit. Note that a count bias of negative three is reported in Table 1 for the away classification in P2 compared to only two instances of this misclassification reported in the confusion matrix in Figure 3b. This resulted from a single case in which the classifier missed a transition from *away* to *sit* and remained in the *away* state. When the participant returned to the *away* state, this sit-to-away transition was also missed, but it serendipitously resulted in the classifier being correct, even though it missed two transitions. Figure 3c shows the variability in detection across multiple users. For P1, since there was 100% success, there is no variability. For P2, the average number of events captured is close to the expected values of the actual number of events, and the possibility of a misclassification is low.

### 5.3. Agreement and Temporal Tolerance Analysis

To evaluate the system’s temporal fidelity, we analyzed the relationship between measurement error (Bland–Altman) and event detection sensitivity (Time Tolerance). This combined analysis determines the optimal error threshold for capturing true positive events.

**Figure 4:**
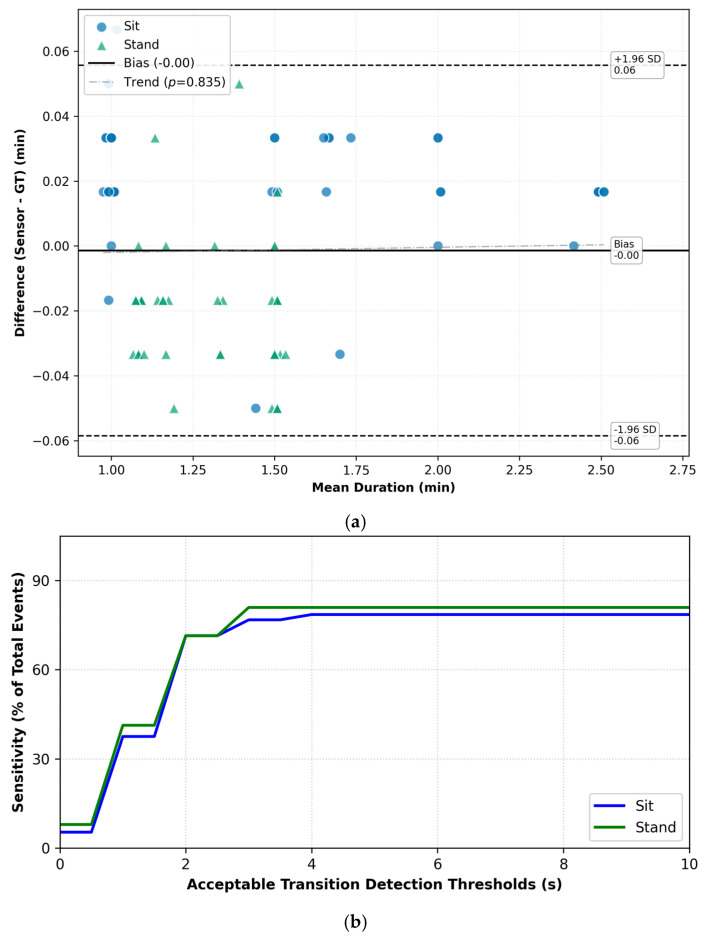
Protocol 1 (**a**) Bland–Altman Agreement; (**b**) Sensitivity vs. Delay Tolerance *δ*.

The Bland–Altman plot (Figure 4a) reveals high agreement with the ground truth in protocol 1, showing negligible mean bias (−0.00 min). The 95% LoA was calculated at ±0.06 min (approximately ±3.6 s).

This measurement variance directly explains the sensitivity trajectory shown in Figure 4b. Detection rates rise sharply between 0.5 and 3 or 4 s and plateau as the time tolerance encompasses the ±3.6-s LoA boundary. This confirms that a *δ_min_* = 4-s window is necessary to account for the inherent latency in detecting random passive static postural shifts. Note that the *δ* in the *x* axis can be increased infinitely to obtain 100% sensitivity in theory, but such delays would be unacceptable, and we stop when successive increments in *δ* do not yield any improvement in sensitivity.

**Figure 5:**
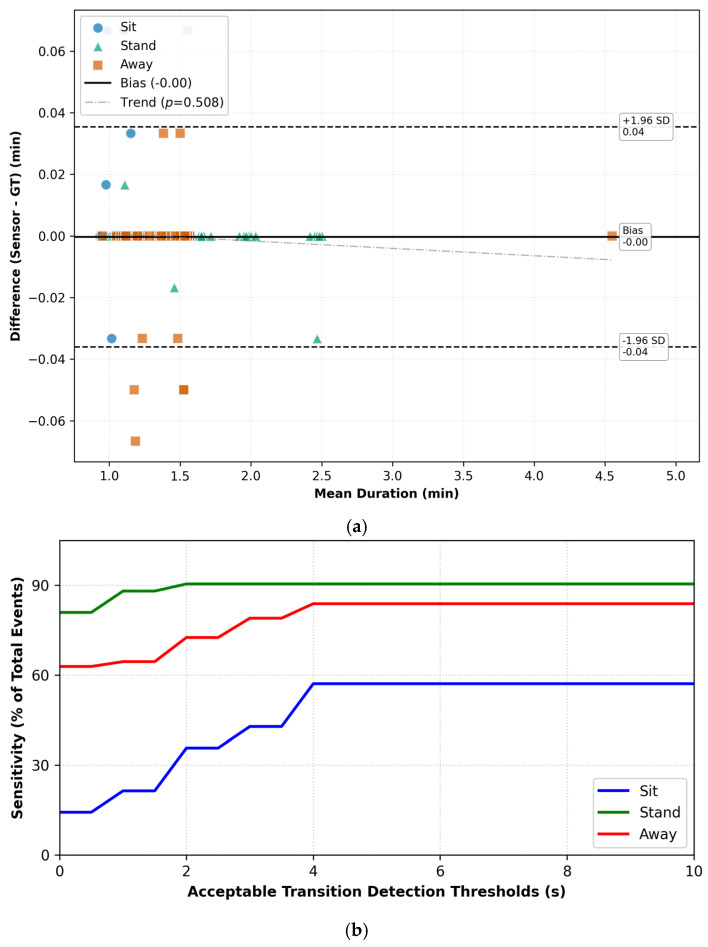
Protocol 2 (**a**) Bland–Altman Agreement; (**b**) Sensitivity vs. Delay Tolerance *δ*.

In protocol 2, agreement remained robust in distinguishing between presence and absence. The Bland–Altman analysis (Figure 5a) indicated a mean bias of −0.00 min with a narrower LOA of ±0.04 min (±2.4 s).

This increased precision is reflected in the corresponding sensitivity curve (Figure 5b), where detection rates for ‘away’ and ‘stand’ events stabilize rapidly. Although the measurement variance was lower than in protocol 1, maintaining the 4-s tolerance threshold maximizes recall without compromising specificity.

In both protocols, regression analysis of the Bland–Altman data showed no significant proportional bias (*p* = 0.835 for P1; *p* = 0.508 for P2), indicating that the sensor’s accuracy remains stable across event durations.

### 5.4. Human Behavior Characteristics

**Figure 6:**
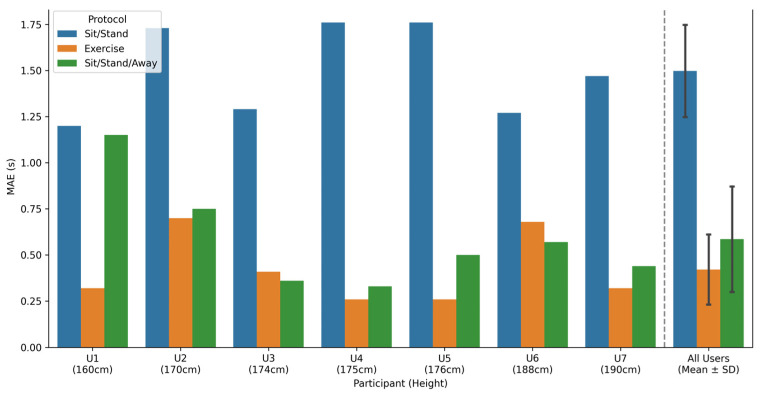
Comparison of MAE across individuals, considering *δ_min_*.

Figure 6 illustrates the MAE for each participant. Sensor temporal accuracy varied by user: Participant 3 (U3) achieved the highest overall accuracy, while Participant 2 (U2) recorded the lowest. Participant height (160–190 cm) showed no significant correlation with these error metrics; however, the small sample size limits the generalizability of this finding. Another observation is that for P3, the MAE is consistently lower than the P1, while P2 and P1 are roughly the same for everyone, except U1.

### 5.5. Computational Load

As the sensor is designed to be low-cost, it does not come with a self-powered processing mechanism. It just connects to the user’s PC via USB and runs on the PC’s power. While power consumption is negligible, the data streamed to the PC must be processed, i.e., the Differential Distance Classifier algorithm must run on the PC as background software. This must not put an excessive load on PCs, as it is meant to be a smooth-running workstation for the corresponding user in the workplace.

With the software written for this lab trial, the CPU utilization was 0.04%. This demonstrates that the algorithm is lightweight and suitable for edge computing (processing on the PC rather than in the cloud), thereby preserving local battery life and reducing bandwidth requirements.

## 6. Discussion

### 6.1. Principal Findings

This study validates a privacy-preserving, edge-computing sensor integrated into a workplace health intervention. The results demonstrate both the device’s performance and the nature of human-device interactions.

#### 6.1.1. Device Performance

The system achieves high fidelity in measuring the aggregate volume of sedentary time, with low count bias and low MAE in all protocols.

Crucially, the 100% sensitivity observed in Protocol 3 (Exercise) demonstrates that the current sampling rate is sufficient to accurately detect movement patterns such as desk squats. This confirms that architecture can distinguish between low-frequency postural shifts and high-frequency gestures, a prerequisite for automated exercise logging.

#### 6.1.2. Human Alertness and Intent

From a human-behavior perspective, we observed that the sensor performed better in P3 than in P1 when *δ_min_* was applied. Although in both cases the sensor correctly classified ‘sit’ and ‘stand’, in P1, where participants were not focused on the activity and were allowed to engage in casual tasks, detection was too slow in about 20% of cases (as shown in Table 1: sensitivity). In the case of P3, where the participants were focused on the rapid sit/stand task within a specific time frame and remained fully focused while doing so, all stand and sit transitions were detected correctly with shorter latency. Even when compared to P2, which also includes sit-and-stand, P3’s performance and accuracy are much better.

### 6.2. Methodological Implications: The Limits of Accelerometry

The results from Protocol 2 empirically demonstrate the “geometric blindness” inherent in standard thigh-worn accelerometry. While the AP3 accurately detected upright posture, its reliance on gravity orientation led to the misclassification of “*away*” events as “*standing*.” This confirms that while wearables remain the criterion standard for total physical activity volume, they are insufficient for establishing the specific contextual awareness required for desk-based interventions.

In contrast, the tested sensor successfully utilized spatial presence to resolve this ambiguity, distinguishing between workstation use and user absence without false positives. Beyond these data-driven advantages, the study suggests that environmental sensing offers a superior modality for longitudinal workplace monitoring. By eliminating the “burden of adherence”, specifically the need for battery management and skin-mounted adhesives, the autonomous nature of the desk-based sensor addresses the high attrition rates often observed in long-term wearable studies [11]. Consequently, this system offers a viable pathway to transition from retrospective data logging to real-time, “just-in-time” biofeedback.

### 6.3. Observed Challenges

While the moving-average filter mitigates high-frequency hardware noise, the 60-s clinical filter fails to accommodate low-frequency behavioral artifacts, such as postural sway. Analysis of the raw data revealed that transient movements cause severe signal fragmentation. For example, during a scripted 90-s standing bout, postural sway generated a brief ‘away’ artifact at the 33-s mark. This interruption fragmented the continuous standing bout into distinct 33-s and 56-s segments. Because the initial 33-s segment fell below the 60-s threshold, the filtering logic classified the valid standing time as noise. The system subsequently appended this discarded time to the preceding sitting bout, logging a 94-s sit and a truncated 57-s stand. This mechanism directly explains the underestimation of time spent present. Furthermore, the ‘chair effect’ occlusion occurred exclusively with a single participant, identifying it as a discrete limitation of the initial static range calibration. To mitigate this in future deployments, the system will utilize an interactive calibration process. This protocol will establish user-specific thresholds by requiring the user to (1) stand and exhibit postural sway, (2) sit and move horizontally, and (3) vacate the workstation while moving the empty chair back and forth.

When the chair back mimicked the geometric profile of a human torso, the system erroneously classified it as a “sit” posture. While this resulted in a conservative estimation of “away” time, it highlights the inherent limitation of distinguishing static objects from static human presence using single-point distance topology alone.

### 6.4. Transforming Interventions: From Passive Logging to Implicit Interaction

The integration of real-time movement recognition alters the HCI paradigm of workplace health intervention. Current systems typically rely on static time-based triggers and self-reported compliance, methods susceptible to “alert fatigue” and reporting bias. The proposed sensor architecture addresses these limitations by enabling adaptive intervention logic. Instead of requiring users to manually report adherence, the system treats physical exercises as inputs, detecting specific movement patterns, such as the squats validated in protocol 3, and automatically logging compliance. This automation reduces the user’s cognitive load and ensures objective data capture. Furthermore, real-time spatial awareness facilitates context-aware prompt scheduling. Whereas timers prompt users indiscriminately, this system detects voluntary “away” states or standing breaks and dynamically resets the prompt timer. By reserving notifications for periods of prolonged sedentary behavior, the system preserves alert salience and mitigates user annoyance.

### 6.5. Limitations

While this laboratory validation confirmed that the proposed system successfully classified sitting, standing, and away states, demonstrating unique usage over the activPAL, transitioning the system to office environments introduces unstructured variables. Future field deployments must account for diverse workstation topologies, multi-monitor configurations that alter typical viewing distances, unconventional user postures, and the complexities of open-plan workplaces.

The algorithmic challenges observed in Section 6.3 dictate the number of software iterations required. Future software iterations will implement interactive angle calibration routines to compensate for inter-subject variables, such as user height and monitor orientation, thereby maximizing the sensor’s effective detection range. To mitigate the “chair effect” occlusion, future deployments will implement an interactive calibration mode to generate user-specific workspace profiles and establish baseline depth thresholds.

Additionally, the signal conditioning window (ω=8), optimized for this specific laboratory setup, requires reevaluation. Future work will analyze the impact of varying ω on detection sensitivity across different desk and chair configurations.

### 6.6. Future Action Identification

Fast time-series data sampled at 3–10 Hz can be analyzed as a multivariate temporal signal to identify characteristic behavioral patterns associated with specific user actions. With two distance streams directed toward the user, the system captures coordinated spatial–temporal variations in body position, where each action, sitting, standing, or moving away from the monitored area, produces a distinct temporal trajectory across the paired sensors. Humans typically exhibit repeatable movement patterns when transitioning between these states, as reflected in the rate of change in distance, the relative timing between the two sensors, and the magnitude of displacement. By analyzing the signals within short sliding windows (e.g., 2–5 s, 20–50 samples per channel), temporal descriptors such as first-order derivatives, signal slope, inter-sensor differential, cross-correlation, variance, and temporal persistence can be extracted. These descriptors encode motion dynamics and spatial relationships between the sensors, forming a discriminative representation of the underlying action pattern while reducing sensitivity to static positional differences among users.

The issue of height should not significantly impact the identified patterns. Also, as the sensor can be adjusted for the angle between sensors, it is possible to configure each sensor to obtain specific sit-and-stand patterns.

With labeled datasets capturing multiple users and repeated behavioral transitions, these movement patterns can be statistically modeled and learned using compact machine learning models. For real-time deployment on resource-constrained microcontrollers, the classification stage can be implemented using lightweight TinyML approaches, quantized 1-D convolutional neural networks operating on fixed-length temporal windows, which are trained offline and deployed via frameworks such as TensorFlow Lite for Microcontrollers. These models capture local temporal dependencies while maintaining a small computational and memory footprint suitable for embedded inference. The runtime pipeline typically includes continuous window buffering, feature normalization, and periodic model evaluation, followed by temporal smoothing mechanisms, such as majority voting or confidence averaging, to reduce false detections caused by sensor noise or incidental movement. This enables robust, low-latency on-device classification of human activity states from low-frequency distance sensing streams.

## 7. Conclusions

This study validates a low-cost, privacy-preserving IoT sensor that quantifies sedentary behavior and recognizes therapeutic exercise gestures. By matching the duration accuracy of video analysis for stationary tasks and outperforming wearables in detecting desk-based exercise, this architecture supports a new generation of “just-in-time” adaptive interventions. By replacing passive monitoring with real-time gesture recognition, the system enables automated exercise logging and context-aware prompt scheduling, offering a scalable solution to the adherence barriers plaguing modern office health interventions.

## Figures and Tables

**Table 1:** Duration and event detection performance of the ToF sensor.

Protocol	State	Mean Difference (min) [95% CI]	MAE (Sec ± SD)	Sensitivity (%)	*δ_min_* (s)	Count Bias
1	Sit	0.02 [0.01, 0.03]	1.57 ± 0.82	78.6	3	0
	Stand	−0.02 [−0.03, −0.01]	1.51 ± 0.83	81.0	4	0
2	Sit	0.01 [−0.04, 0.05]	2.00 ± 1.60	71.4	4	−1
	Stand	−0.00 [−0.01, 0.00]	0.13 ± 0.41	90.5	2	−2
	Away	−0.00 [−0.01, 0.00]	0.67 ± 1.26	87.3	4	−3
3	Exercise	—	—	100.0	2	0

**Table 2:** Classification performance metrics (%) for Protocols 1 and 2.

Class	Precision	Recall (Sensitivity)	Specificity	*F*_1_-Score
Sit (P1)	100.00	100.00	100.00	100.00
Stand (P1)	100.00	100.00	100.00	100.00
Sit (P2)	81.25	92.86	97.14	86.67
Stand (P2)	100.00	95.24	100.00	97.56
Away (P2)	96.83	96.83	96.43	96.83

## Data Availability

The data presented in this study are not publicly available due to privacy and ethical restrictions regarding the anonymity of human participants. The data presented in this study are available on request from the corresponding author due to (specify the reason for the restriction). The software source code is not publicly available due to intellectual property restrictions.

## Abbreviations

The following abbreviations are used in this manuscript:

AP3	activPAL3
HCI	Human–Computer Interaction
IMU	Inertial Measurement Unit
KNN	K-Nearest Neighbors
LoA	Limits of Agreement
MAE	Mean Absolute Error
MFCC	Mel-Frequency Cepstral Coefficients
RQA	Recurrence Quantification Analysis
SVM	Support Vector Machines
ToF	Time-of-Flight
P1	Protocol 1
P2	Protocol 2
P3	Protocol 3

## Appendix A

### Experimental Protocols

**Table A1:** Protocol 1: Sitting and Standing.

*Order*	*Scripted Action*	*Scripted Duration (s)*
1	Stand	70
2	Sit	60
3	Stand	90
4	Sit	60
5	Stand	90
6	Sit	60
7	Stand	90
8	Sit	60
9	Stand	80
10	Sit	90
11	Stand	65
12	Sit	100
13	Stand	65
14	Sit	120
15	Stand	65
16	Sit	150
17	Stand	90

**Table A2:** Protocol 2: Sitting, Standing, and Away.

*Order*	*Scripted Action*	*Scripted Duration (s)*
1	Away	70
2	Stand	70
3	Away	90
4	Sit	70
5	Away	90
6	Stand	60
7	Away	90
8	Sit	60
9	Away	80
10	Stand	90
11	Away	65
12	Stand	100
13	Away	65
14	Stand	120
15	Away	65
16	Stand	150
17	Away	90

**Table A3:** Protocol 3: Exercise.

*Order*	*Scripted Action*	*Scripted Duration (s)*
1	Stand	3
2	Sit	3
3	Stand	3
4	Sit	3
5	Stand	3
6	Sit	3
7	Stand	3
8	Sit	3
9	Stand	3
10	Sit	3
11	Stand	3
12	Sit	3
13	Stand	3
14	Sit	3
15	Stand	3
16	Sit	3
17	Stand	3
18	Sit	3
19	Stand	3
20	Sit	3

## References

[1] Clemes S.A., O’Connell S.E., Edwardson C.L. (2014). Office Workers’ Objectively Measured Sedentary Behavior and Physical Activity during and Outside Working Hours. J. Occup. Environ. Med..

[2] Chandrasekaran B., Ganesan T.B. (2021). Sedentarism and Chronic Disease Risk in COVID 19 Lockdown—A Scoping Review. Scott. Med. J..

[3] Liang Z., Zhang M., Wang C., Yuan Y., Liang J. (2022). Association between Sedentary Behavior, Physical Activity, and Cardiovascular Disease-Related Outcomes in Adults—A Meta-Analysis and Systematic Review. Front. Public Health.

[4] Zhai L., Zhang Y., Zhang D. (2015). Sedentary Behaviour and the Risk of Depression: A Meta-Analysis. Br. J. Sports Med..

[5] Majumdar P., Biswas A., Sahu S. (2020). COVID-19 Pandemic and Lockdown: Cause of Sleep Disruption, Depression, Somatic Pain, and Increased Screen Exposure of Office Workers and Students of India. Chronobiol. Int..

[6] Bakker E.A., Hartman Y.A.W., Hopman M.T.E., Hopkins N.D., Graves L.E.F., Dunstan D.W., Healy G.N., Eijsvogels T.M.H., Thijssen D.H.J. (2020). Validity and Reliability of Subjective Methods to Assess Sedentary Behaviour in Adults: A Systematic Review and Meta-Analysis. Int. J. Behav. Nutr. Phys. Act..

[7] Chastin S.F.M., Dontje M.L., Skelton D.A., Čukić I., Shaw R.J., Gill J.M.R., Greig C.A., Gale C.R., Deary I.J., Der G. (2018). Systematic Comparative Validation of Self-Report Measures of Sedentary Time against an Objective Measure of Postural Sitting (activPAL). Int. J. Behav. Nutr. Phys. Act..

[8] Pedersen S.J., Kitic C.M., Bird M.-L., Mainsbridge C.P., Cooley D. (2016). Is Self-Reporting Workplace Activity Worthwhile? Validity and Reliability of Occupational Sitting and Physical Activity Questionnaire in Desk-Based Workers. BMC Public Health.

[9] Craig C.L., Marshall A.L., Sjöström M., Bauman A.E., Booth M.L., Ainsworth B.E., Pratt M., Ekelund U., Yngve A., Sallis J.F. (2003). International Physical Activity Questionnaire: 12-Country Reliability and Validity. Med. Sci. Sports Exerc..

[10] Prati A., Shan C., Wang K.I.-K. (2019). Sensors, Vision and Networks: From Video Surveillance to Activity Recognition and Health Monitoring. J. Ambient. Intell. Smart Environ..

[11] Steeves J.A., Bowles H.R., Mcclain J.J., Dodd K.W., Brychta R.J., Wang J., Chen K.Y. (2015). Ability of Thigh-Worn ActiGraph and activPAL Monitors to Classify Posture and Motion. Med. Sci. Sports Exerc..

[12] Maiti A., Ye A., Schmidt M., Pedersen S. (2023). A Privacy-Preserving Desk Sensor for Monitoring Healthy Movement Breaks in Smart Office Environments with the Internet of Things. Sensors.

[13] Maiti A., Ward V., Hilliard A., Ye A., Pedersen S.J. (2025). Design Principles of a Flat-Pack Electronic Sensor Kit with Intelligent User Interface Calibrations: A Case Study of Monitoring Sedentary Behavior in Workplace. Appl. Sci..

[14] Cooley D., Pedersen S. (2013). A Pilot Study of Increasing Nonpurposeful Movement Breaks at Work as a Means of Reducing Prolonged Sitting. J. Environ. Public Health.

[15] Mainsbridge C.P., Cooley P.D., Fraser S.P., Pedersen S.J. (2014). The Effect of an E-Health Intervention Designed to Reduce Prolonged Occupational Sitting on Mean Arterial Pressure. J. Occup. Environ. Med..

[16] Mainsbridge C.P., Cooley D., Dawkins S., de Salas K., Tong J., Schmidt M.W., Pedersen S.J. (2020). Taking a Stand for Office-Based Workers’ Mental Health: The Return of the Microbreak. Front. Public Health.

[17] Carter S.E., Draijer R., Maxwell J.D., Morris A.S., Pedersen S.J., Graves L.E.F., Thijssen D.H.J., Hopkins N.D. (2020). Using an E-Health Intervention to Reduce Prolonged Sitting in UK Office Workers: A Randomised Acceptability and Feasibility Study. Int. J. Environ. Res. Public Health.

[18] Oliver H., Thomas O., Neil R., Copeland R.J., Moll T., Chadd K., Jukes M.J., Quartermaine A. (2024). A Longitudinal Study Combining the Double Diamond Framework and Behavior Change Wheel to Co-Create a Sedentary Behavior Intervention in Police Control Rooms. J. Public Health.

[19] Wu C., Wang B., Shen G. (2022). Unobtrusive Monitoring of Sedentary Behaviors with Fusion of Bluetooth and Ballistocardiogram Signals. Methods.

[20] Shum L.C., Faieghi R., Borsook T., Faruk T., Kassam S., Nabavi H., Spasojevic S., Tung J., Khan S.S., Iaboni A. (2022). Indoor Location Data for Tracking Human Behaviours: A Scoping Review. Sensors.

[21] Biswas S., Harrington B., Hajiaghajani F., Wang R. Contact-Less Indoor Activity Analysis Using First-Reflection Echolocation. Proceedings of the 2016 IEEE International Conference on Communications (ICC).

[22] Ryde G.C., Gilson N.D., Suppini A., Brown W.J. (2012). Validation of a Novel, Objective Measure of Occupational Sitting. J. Occup. Health.

[23] Alemdar H., Ersoy C. (2010). Wireless Sensor Networks for Healthcare: A Survey. Comput. Netw..

[24] Zhou Y., Hua Y., Liu J. (2021). Study Workplace Space Occupancy: A Review of Measures and Technologies. J. Facil. Manag..

[25] Weizman Y., Tan A.M., Fuss F.K. (2023). The Use of Wearable Devices to Measure Sedentary Behavior during COVID-19: Systematic Review and Future Recommendations. Sensors.

[26] Chang V., Eniola R.O., Golightly L., Xu Q.A. (2023). An Exploration into Human–Computer Interaction: Hand Gesture Recognition Management in a Challenging Environment. SN Comput. Sci..

[27] Holtermann A., Schellewald V., Mathiassen S.E., Gupta N., Pinder A., Punakallio A., Veiersted K.B., Weber B., Takala E.-P., Draicchio F. (2017). A Practical Guidance for Assessments of Sedentary Behavior at Work: A PEROSH Initiative. Appl. Ergon..

[28] Kańtoch E. (2018). Recognition of Sedentary Behavior by Machine Learning Analysis of Wearable Sensors during Activities of Daily Living for Telemedical Assessment of Cardiovascular Risk. Sensors.

[29] Sinha V.K., Patro K.K., Pławiak P., Prakash A.J. (2021). Smartphone-Based Human Sitting Behaviors Recognition Using Inertial Sensor. Sensors.

[30] Matthews C.E., Saint-Maurice P., Freeman J.R., Hayes H.A., Shreves A.H., Doherty A., Hyde E.T., Ylarregui K., Jones R.R., Keadle S.K. (2025). Performance Evaluation of Algorithms to Estimate Daily Sedentary Time Using Wrist-Worn Sensors in Free-Living Adults. J. Meas. Phys. Behav..

[31] Boudet G., Chausse P., Thivel D., Rousset S., Mermillod M., Baker J.S., Parreira L.M., Esquirol Y., Duclos M., Dutheil F. (2019). How to Measure Sedentary Behavior at Work?. Front. Public Health.

[32] Zahra R., Shehzadi A., Sharif M.I., Karim A., Azam S., De Boer F., Jonkman M., Mehmood M. (2023). Camera-Based Interactive Wall Display Using Hand Gesture Recognition. Intell. Syst. Appl..

[33] Sideridis V., Zacharakis A., Tzagkarakis G., Papadopouli M. GestureKeeper: Gesture Recognition for Controlling Devices in IoT Environments. Proceedings of the 2019 27th European Signal Processing Conference (EUSIPCO).

[34] Sun Y., Fei T., Li X., Warnecke A., Warsitz E., Pohl N. (2020). Real-Time Radar-Based Gesture Detection and Recognition Built in an Edge-Computing Platform. IEEE Sens. J..

[35] Yadav S., Jain S. Gesture Recognition System for Human-Computer Interaction Using Computer Vision. Proceedings of the 2024 11th International Conference on Reliability, Infocom Technologies and Optimization (Trends and Future Directions) (ICRITO).

[36] Meng Z., Zhang M., Guo C., Fan Q., Zhang H., Gao N., Zhang Z. (2020). Recent Progress in Sensing and Computing Techniques for Human Activity Recognition and Motion Analysis. Electronics.

[37] Hammad A.S., Tajammul A., Dergaa I., Al-Asmakh M. (2025). Machine Learning Applications in the Analysis of Sedentary Behavior and Associated Health Risks. Front. Artif. Intell..

[38] Buffey A.J., Herring M.P., Langley C.K., Donnelly A.E., Carson B.P. (2022). The Acute Effects of Interrupting Prolonged Sitting Time in Adults with Standing and Light-Intensity Walking on Biomarkers of Cardiometabolic Health in Adults: A Systematic Review and Meta-Analysis. Sports Med.

[39] Harrington D.M., Welk G.J., Donnelly A.E. (2011). Validation of MET Estimates and Step Measurement Using the ActivPAL Physical Activity Logger. J. Sports Sci..

[40] Sokolova M., Lapalme G. (2009). A Systematic Analysis of Performance Measures for Classification Tasks. Inf. Process. Manag..

